# Supplementary material for: Circulating miRNA Correlates with Lipid Profile and Disease Activity in Psoriatic Arthritis, Rheumatoid Arthritis, and Ankylosing Spondylitis Patients
Source: Biomedicines. 2022 Apr 13;10(4):893. doi: 10.3390/biomedicines10040893 (PMC9024741; doi:10.3390/biomedicines10040893)
Supplement: Supplementary file 1 [file biomedicines-10-00893-s001.zip › biomedicines-1663850-supplementary.pdf]

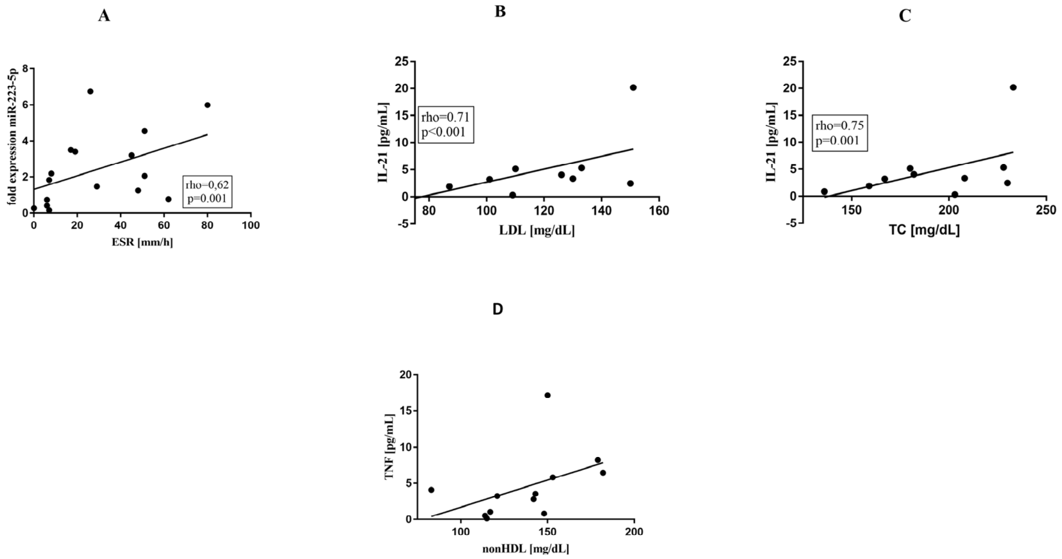

Supplementary Figure S1. Correlation analyses in PsA (A) and RA (B-D) group of patients.

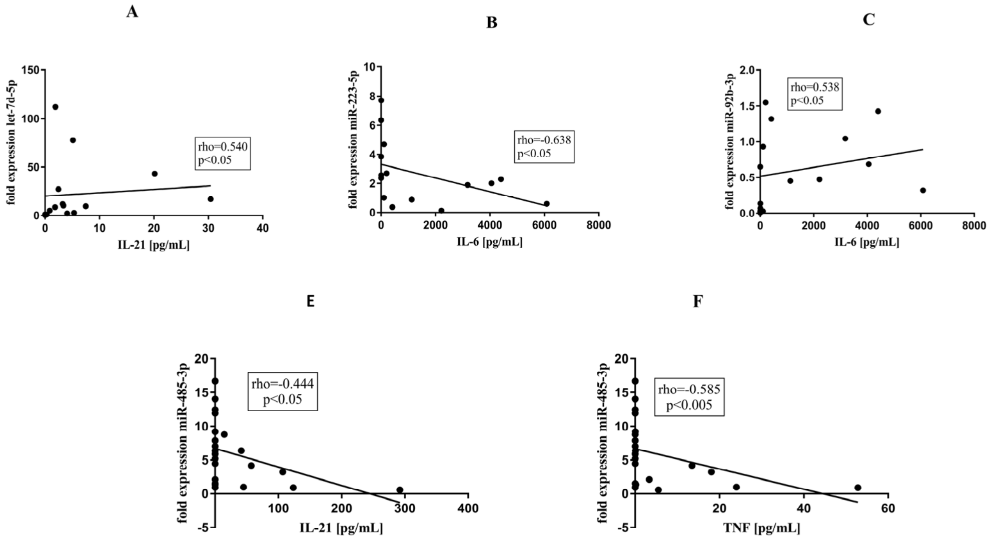

Supplementary Figure S2. Correlation analyses of serum cytokine levels with miR expression in RA group of patients (A-C) and AS group of patients (E-F).

Variables	AUC	Standarized error	P	Confidence Intervals		Threshold
				Lower Bound	Upper Bound	
**mir485-3**	**0,65**	**0,08**	**0,05**	**0,50**	**0,80**	**1,00**
mir92	0,64	0,08	0,09	0,48	0,81	
**miR26-a-2-3**	**0,82**	**0,07**	**0,00**	**0,68**	**0,95**	**6,00**
miR10-b-5p	0,66	0,10	0,11	0,47	0,85	
mir146-3-p	0,66	0,09	0,10	0,47	0,84	
mir-233	0,60	0,09	0,25	0,43	0,78	
mir-222	0,41	0,08	0,29	0,25	0,58	
mir-let7d	0,51	0,09	0,92	0,34	0,68	

Table S1A ROC curves for miRs distinguishing PsA

Variables	AUC	Standarized error	P	Confidence Intervals		Threshold
				Lower Bound	Upper Bound	
**mir485-3**	**0,72**	**0,06**	**0,00**	**0,60**	**0,84**	**5,10**
mir92	0,50	0,07	0,96	0,36	0,65	
**miR26-a-2-3**	**0,66**	**0,06**	**0,01**	**0,54**	**0,79**	**2,10**
miR10-b-5p	0,62	0,06	0,06	0,49	0,75	
mir146-3-p	0,47	0,07	0,68	0,34	0,61	
mir-233	0,47	0,07	0,64	0,33	0,60	
mir-222	0,60	0,07	0,15	0,46	0,73	
mir-let7d	0,40	0,07	0,11	0,27	0,52	

Table S1B ROC curves for miRs distinguishing AS

Variables	AUC	Standarized error	P	Confidence Intervals		Threshold
				Lower Bound	Upper Bound	
mir485-3	0,47	0,08	0,69	0,32	0,62	
mir92	0,31	0,08	0,02	0,15	0,46	
miR26-a-2-3	0,37	0,07	0,07	0,22	0,51	
miR10-b-5p	0,27	0,08	0,01	0,11	0,43	

**mir146-3-p**	**0,65**	**0,07**	**0,03**	**0,51**	**0,79**	**1,20**
mir-233	0,58	0,09	0,39	0,40	0,75	
mir-222	0,62	0,09	0,16	0,45	0,79	
**mir-let7d**	**0,81**	**0,07**	**0,00**	**0,67**	**0,96**	**8,1**

Table S1C ROC curves for miRs distinguishing RA
